# MiR-214-3p targets Ras-related protein 14 (RAB14) to inhibit cellular migration and invasion in esophageal Cancer cells

**DOI:** 10.1186/s12885-022-10304-0

**Published:** 2022-12-05

**Authors:** Pornima Phatak, Whitney M. Burrows, Timothy Michael Creed, Mariam Youssef, Goo Lee, James M. Donahue

**Affiliations:** 1 Birmingham Veterans Affairs Medical Center, Birmingham, AL USA; 2 Department of Surgery, University of Alabama at Birmingham, Birmingham, AL USA; 3 Baltimore Veterans Affairs Medical Center, Baltimore, MD USA; 4 Department of Surgery, University of Maryland School of Medicine, Baltimore, MD USA; 5 Center for Stem Cell Biology & Regenerative Medicine, University of Maryland School of Medicine, Baltimore, MD USA; 6 Department of Pathology, University of Alabama at Birmingham, Birmingham, AL USA

**Keywords:** RAB14, Esophageal Cancer, Micro RNA

## Abstract

**Background:**

MicroRNA (miR)-214-3p is emerging as an important tumor suppressor in esophageal cancer. In this study, we examined the interaction between miR-214-3p and RAB14, a membrane trafficking protein shown to exert oncogenic functions in other malignancies, in esophageal cancer cells.

**Methods:**

Studies were performed in a human esophageal epithelial cell line and a panel of esophageal cancer cell lines, as well in human specimens. MiR-214-3p expression was measured by digital PCR. Biotinylated RNA pull-down and luciferase reporter assays assessed binding. The xCELLigence RTCA system measured cell migration and invasion in real time. A lentiviral expression vector was used to create an esophageal cancer cell line stably expressing miR-214-3p.

**Results:**

MiR-214-3p expression was decreased in esophageal cancer cell lines and human specimens compared to non-malignant controls. RAB14 mRNA stability and protein expression were decreased following miR-214-3p overexpression. Binding between miR-214-3p and RAB14 mRNA was observed. Either forced expression of miR-214-3p or RAB14 silencing led to a marked decrease in cellular migration and invasion. Esophageal cancer cells stably expressing miR-214-3p demonstrated decreased growth in a subcutaneous murine model.

**Conclusions:**

These results further support the tumor-suppressive role of miR-214-3p in esophageal cancer cells by demonstrating its ability to regulate RAB14 expression.

**Supplementary Information:**

The online version contains supplementary material available at 10.1186/s12885-022-10304-0.

## Background

From 2012 to 2018, the incidence of esophageal cancer increased by 25% worldwide [1, 2]. With an overall 5-year survival rate of approximately 20%, esophageal cancer is the sixth leading cause of cancer death worldwide, and the seventh leading cause of cancer death in men in the United States [2, 3]. Most esophageal cancers demonstrate limited sensitivity to current chemotherapeutic regimens. Despite a recent thorough analysis of the esophageal cancer genome, currently no targetable driver mutations have been identified for the treatment of esophageal cancer [4, 5]. Alternative molecular strategies are required to identify targets that can serve therapeutic and predictive roles to improve outcomes for esophageal cancer patients given the rising prevalence of this deadly disease.

MicroRNAs (miRs) are small, non-coding, highly conserved RNAs, which are recognized as important post-transcriptional regulators of gene expression in cancer cells [6]. MiRs, which have been shown to be dysregulated in multiple malignancies, can act as both oncogenes and tumor suppressors by affecting critical cellular processes such as proliferation, apoptosis, and invasiveness [7]. Previous studies comparing miR expression in human esophageal cancer samples to matched normal esophageal epithelium have demonstrated distinctive patterns of miR expression that can distinguish malignant from normal tissue [8, 9]. More recent efforts have identified specific miRs with prognostic capabilities in esophageal squamous cell cancer [10]. The most striking data attesting to the important role of miRs in esophageal cancer comes from a recent study describing the ability of germline knockout of miR-31 to eliminate the development of esophageal squamous cell cancer in a zinc-deficient rat model [11].

We have previously compared global miR expression in a human esophageal epithelial cell line (hESO) to the human esophageal squamous cell cancer lines TE7 and TE10 [12]. In this study, miR-214-3p was downregulated by approximately 3 log-fold in both TE7 and TE10 cells compared to hESO cells. We demonstrated that miR-214-3p regulated expression of both survivin, an anti-apoptotic protein, and CUGBP1, an RNA-binding protein which enhances survivin expression. Restoration of miR-214-3p expression in TE7 and TE10 cells markedly downregulated expression of both survivin and CUG-BP1, resulting in significantly increased sensitivity to cisplatin-induced apoptosis in these cells.

Due to inherent incompleteness in pairing between the seed sequence in miRs and their target mRNAs, an individual miR may regulate multiple targets [6]. This allows for the possibility that an individual miR could serve as a master regulator in cancer cells by coordinating the regulation of multiple, critical oncogenic targets. We searched miR-target prediction programs in order to identify other potential targets for miR-214-3p in esophageal cancer cells that may influence oncogenic functions in addition to apoptosis. We found that miR-214-3p is predicted to bind the mRNA encoding RAB14 protein with high affinity. RAB14 is a member of the ras-associated binding protein family of low molecular mass GTPases that are involved in membrane trafficking and has been shown to be overexpressed in several malignancies. The current study was designed to assess the expression levels of miR-214-3p and RAB14 in esophageal cancer cell lines as well as in human esophageal cancer specimens. Functional, binding, and phenotypic assays were performed to characterize the interaction between miR-214-3p and RAB14 mRNA in esophageal cancer cells.

## Methods

### Cell culture and reagents

The human esophageal squamous cancer cell line TE7 and esophageal epithelial cell line hESO were obtained and maintained as previously described [12]. The human esophageal adenocarcinoma cancer cell lines FLO-1 and SK-GT-4 were purchased from European Collection of Authenticated Cell Culture (England, UK). SK-GT-4 cells were cultured in RPMI and FLO-1 cells were cultured in DMEM medium (Thermo-fisher, Carlsbad, CA, USA) supplemented with 10% heat-inactivated FBS (Invitrogen, Carlsbad, CA, USA). All cells were maintained in a 37 °C incubator with 5% CO2 humidified air.

### Transfection

Transfection of cells was done as previously described [12]. Briefly, cells were seeded in 6 cm dishes at density of 0.7–1.0. × 10^6^, a day prior to transfection. For miR transfections, pre-miR-214-3p (50 nM), anti-miR-214-3p (25 nM), or control miR (25 nM) (Ambion, Austin, TX, USA) were diluted in 500 μl Opti-MEM I (Invitrogen, Carlsbad, CA, USA) containing 5 μl Lipofectamine RNAiMAX (Invitrogen, Carlsbad, CA, USA). After 15 min incubation at room temperature, the complex was added to the cells in a final volume of 5 ml of fresh medium. In overexpression experiments, 2.5 μg RAB14 expression plasmid (Kind gift from Dr. Civin) was used. Silencing of RAB14 was done using pooled 80 pmol si-RAB14-RNA (Santa Cruz, CA, USA).

### MiR-214-3p overexpressing stable cell lines

FLO-1 cells were plated in a 12-well plate (50 × 10^3^/well) and transduced using polybrene (8 μg/ml) either with 100 μl control lentivirus or hsa-miR-214 lentivirus (Biosettia, San Diego, CA, USA) in a total volume of 1 mL the next day. Medium was replaced after 24 h with Puromycin (1 μg/ml, Sigma, Saint Louis, MO, USA) as a selection marker. Clones were selected over a period of 10 days with puromycin, before transferring to a 96 well plate in 100 μl of final volume to isolate single clones. Clones were then transferred to 24 and 6 well plates, respectively. Single clones were screened by q-PCR for miR-214-3p and RAB14 mRNA expression.

### Reverse transcription (RT) and quantitative real-time PCR (q-PCR) analyses

RT was done using the q-script cDNA synthesis kit (Quantabio, Beverly, MA, USA) as per the manufacturer’s instructions. Q-PCR experiments were performed using the Step-One Plus system. All the PCR reactions were performed in triplicate with specific (RAB14, RAB15, miR-214-3p, U6, and GAPDH) TaqMan primers and probes (Thermo-fisher, Foster City, CA, USA). The levels of RAB14 and RAB15 were normalized with GAPDH. For miR experiments, normalization was accomplished using small nuclear RNA U6.

### Esophageal cancer specimens

Samples of esophageal tumor as well as adjacent non-malignant epithelium with no evidence of Barrett’s esophagus were obtained at the time of esophagectomy from patients enrolled in an Institutional Reviewed Board approved protocol (# HP00049069) at the Baltimore VA Medical Center and the University of Maryland Medical Center. The study met all requirements of the Declaration of Helsinki and informed consent was obtained from all patients. All patients had esophageal adenocarcinoma and received neoadjuvant chemoradiotherapy prior to surgical resection. Half of each sample was snap-frozen in liquid nitrogen and the other half placed in formalin for later histologic analysis. Frozen tissues were grinded to a fine powder using mortar and pestle. Lysis buffer was added to tissue powder and lysates were homogenized using QIAshredder (QIAGEN, Valencia, CA). Total RNA was extracted from homogenized lysates using miRNeasy Mini Kit (QIAGEN, Valencia, CA).

### Immunoblotting

Twenty micrograms of protein were resolved on 10% SDS-PAGE gels (Bio-Rad Laboratories, Hercules, CA, USA) and transferred onto PVDF membranes (GE Healthcare, Piscataway, NJ, USA). After transfer, membranes were blocked in 5% nonfat milk in TBST and incubated with anti-human RAB14 and anti-GAPDH antibodies (Santa Cruz, CA, USA) overnight at 4 °C followed by horseradish peroxidase-conjugated anti-mouse or anti-rabbit (Santa Cruz, Dallas, TX, USA) immunoglobulin secondary antibodies for 1 hour at room temperature. Signal was detected by Chemiluminescence Reagent (PerkinElmer, Waltham, MA) and visualized by autoradiography. Dilutions were 1:2000 for primary antibodies and 1:4000 for secondary antibodies. Signal intensity was quantified using Image Lab quantification software (Bio-Rad, Hercules, CA, USA). All the original blots are included as supplemental figures.

### Bioinformatics

Two software programs, TargetScan Human (http://www.targetscan.org) and miRDB (http://mirdb.org/miRDB) were used to identify potential target genes for miR-214-3p.

### mRNA stability

Stability assays for RAB14 mRNA were done as reported previously [12]. Briefly, following transfection of pre- or anti-miR-214-3p as described above, medium containing Actinomycin D (Sigma–Aldrich, St. Louis, MO) at a final concentration of 4 μM was added for specified time points. Total RNA was isolated from each sample and RT and q-PCR reactions were performed in triplicate as described above.

### Droplet digital PCR

Droplet Digital PCR (ddPCR™) was performed using the QX200™ ddPCR™ system (Bio-Rad, Hercules, CA, USA). All reagents were from Bio-Rad. The droplets were generated for each sample PCR reaction mixture using Droplet Generation Oil. Then, C1000™ thermal cycler was used with cycling conditions as follows: 95 °C for 10 minutes, followed by 40 cycles at 94 °C for 30 seconds, then, 60 °C for 1 min, followed by 98 °C for 10 minutes. Plate was then transferred to the QX200™ Droplet Reader and the data were analyzed using QuantaSoft™ Software version 1.7.

### Biotin-labeled pull-down assays

Biotinylated (Dharmacon, Lafayette, CO, USA) pull-down assay was performed as described earlier [12]. Briefly, 1 × 10^6^ TE7 cells were transfected with biotin-labelled miR-214-3p or control miR at a final concentration of 50 nM for 48 h. Whole cell lysates were incubated at 4 °C overnight with 50 μl/sample of streptavidin-Dyna beads (Invitrogen, Carlsbad, CA, USA) coated with yeast tRNA (Ambion, Austin, TX, USA). Next day, beads were washed thoroughly and RNA was isolated using TRIzol (Invitrogen) and standard chloroform-isopropanol method and then subjected to PCR as explained above.

### Luciferase reporter assay

Luciferase reporter constructs were prepared as previously described [12]. For RAB14 (NM_016322.3), individual luciferase reporter constructs were generated that contained either one or both predicted miR-214-3p binding sites in 3 separate 3’UTR fragments. The inserts were amplified by PCR and individual fragments were sub-cloned into a SacI and Xba1 (New England Bio Labs, Ipswich, MA, USA) digested pmirGLO Dual-Luciferase miRNA target expression vector (Promega, Madison, WI, USA). The constructs containing mutations at the seed sequence binding region of potential binding sites were generated using a site-directed mutagenesis kit (Agilent Technologies, Santa Clara, CA, USA). All primer sequences used to create these constructs are listed in Table 1. Restriction enzyme digestion and DNA sequencing confirmed the orientation and sequence of the constructs. For luciferase activity assay, 2 × 10^5^ TE7 cells/well were plated onto 12-well cell culture plates and co-transfected as described above with luciferase reporter constructs (10 ng) and pre-miR-214-3p (50 nM) for 36 hours. Luciferase activity was measured using the Dual Luciferase Reporter Assay kit (Promega), as per manufacturer’s protocol. Levels of firefly luciferase activity were normalized to Renilla luciferase activity.

**Table 1:** Primer sequences used to generate reporter constructs

SN	Name	Sequence	Region	Cutting Site
1	pmiR-GLO-RAB14-both-F	TGGAAA**GAGCTC**CTAGCATCAG	1989–3048	SacI
2	pmiR-GLO-RAB14-both-R	**TCTAGA**CAAGCCGCAGTCTTTAGTTTGT	1989–3048	XbaI
3	pmiR-GLO-RAB14-BS1-R	**TCTAGA**ACAAGCGCTGAAACACCTTT	1989–2423	XbaI
4	pmiR-GLO-RAB14-BS2-F	**GAGCTCT**CAGCGCTTGTGCTGATACA	2413–3072	SacI
5	pmiR-GLO-RAB14-BS2-R	**TCTAGA**GGGAGTCAGGGTATTGCACC	2413–3072	XbaI
6	pmiR-GLO-RAB14-BS1-Del-F	GCACTGTTGCTTACCTGTTTTCTTAACTGTTCTTG	1989–2423	N/A
7	pmiR-GLO-RAB14-BS1-Del-R	CAAGAACAGTTAAGAAAACAGGTAAGCAACAGTGC	1989–2423	N/A
8	pmiR-GLO-RAB14-BS2-Del-F	CCAGGGACCATGACCTGGTGTGTGTGTATATTTAC	2413–3072	N/A
9	pmiR-GLO-RAB14-BS2-Del-R	GTAAATATACACACACACCAGGTCATGGTCCCTGG	2413–3072	N/A

### Cell proliferation assay (MTT assay)

Cells were transfected for 1–5 days with pre-miR-214-3p, then were incubated with 3-(4–5-dimehtylthiazol-2-yl)-2, 5-diphenyltetrazolium bromide (MTT, 5 mg/ml) (Sigma, Saint Louis, MO, USA) for ~ 4 h at 37 °C. Subsequently, the supernatant was replaced with dimethyl sulphoxide to dissolve formazan crystal residues and corresponding optical densities (OD) were measured at 550 nm. OD for control cells was set at 100%.

### Migration and invasion assays

hESO, TE7, FLO-1 and SK-GT-4 cells were transfected either with anti- or pre-miR-214-3p or RAB14 plasmid for 48 h. At that time, cells were harvested and 40–80 × 10^3^ cells / well were subjected to real time migration and invasion analysis using xCELLigence RTCA system (ACSE Biosciences, San Diego, CA, USA).

### Animal studies

Six week old female NRG mice were injected with either FLO-1 wild-type cells or FLO-1 cells stably overexpressing miR-214-3p (5 × 10^6^ cells per mouse in 500 μl PBS). Day 0 was considered when tumors measured 500–700 mm^3^. Mice (*n* = 5) from each group were euthanized at days 0, 8, 15 and 30. Tumors were harvested and weighed at these time points. Tumors harvested at day 30 were then ground and homogenized for extraction of RNA as described for the human esophageal cancer specimens. In a separate experiment, tumor size was measured twice a week in each cohort over a period of 30 days. The study was approved by the University of Maryland and Baltimore VA Medical Center IACUC (# 1016009) and conforms to ARRIVE guidelines.

### Immunohistochemical Stainin

Five micron paraffin sections from human esophageal tumors were baked overnight at 60 °C, then de paraffinized in 3 changes of xylene and hydrated using graded concentrations of ethanol to deionized water. The tissue sections were subjected to antigen retrieval by 0**.**01 M sodium citrate buffer (pH 6) in pressure cooker for 5 min (buffer preheated). Following antigen retrieval, all sections were washed gently in deionized water, then transferred in to 0.05 M Tris-based solution in 0.15 M NaCl with 0.1% v/v Triton-X-100, pH 7.6 (TBST). Endogenous peroxidase was blocked with 3% hydrogen peroxide for 15 min. To reduce further nonspecific background staining, slides were incubated with 5% normal goat serum (Sigma, G9023) for 45 min at RT. All slides then were incubated at 4 °C overnight with anti-RAB14 (Proteintech, 15,662–1-AP, Rabbit polyclonal, 1/100 dilution). After washing with TBST, sections were then incubated with the Goat Anti-Rabbit IgG H&L secondary antibody conjugated with HRP (Abcam ab6721, 1:1000.) Vector Laboratories - ImmPACT DAB Peroxidase (HRP) Substrate Kit (SK4105) was used as the chromogen and hematoxylin (no. 7221, Richard-Allen Scientific, Kalamazoo, MI) as the counterstain. Images were captured at Olympus BX51 upright microscope with Retiga Camera and Bioquant Osteo Image analysis Software.

### Statistical analysis

Results are expressed as the means ± S.D. from three independent experiments with minimum three replicates for each experiment. Data derived from multiple determinations were subjected to two-tailed Student’s t test or Wilcoxon-Mann-Whitney test. *P* values < 0.05 were considered statistically significant.

## Results

### Expression of miR-214-3p and RAB14 in esophageal cancer cell lines and human esophageal cancer specimens

**Fig. 1:**
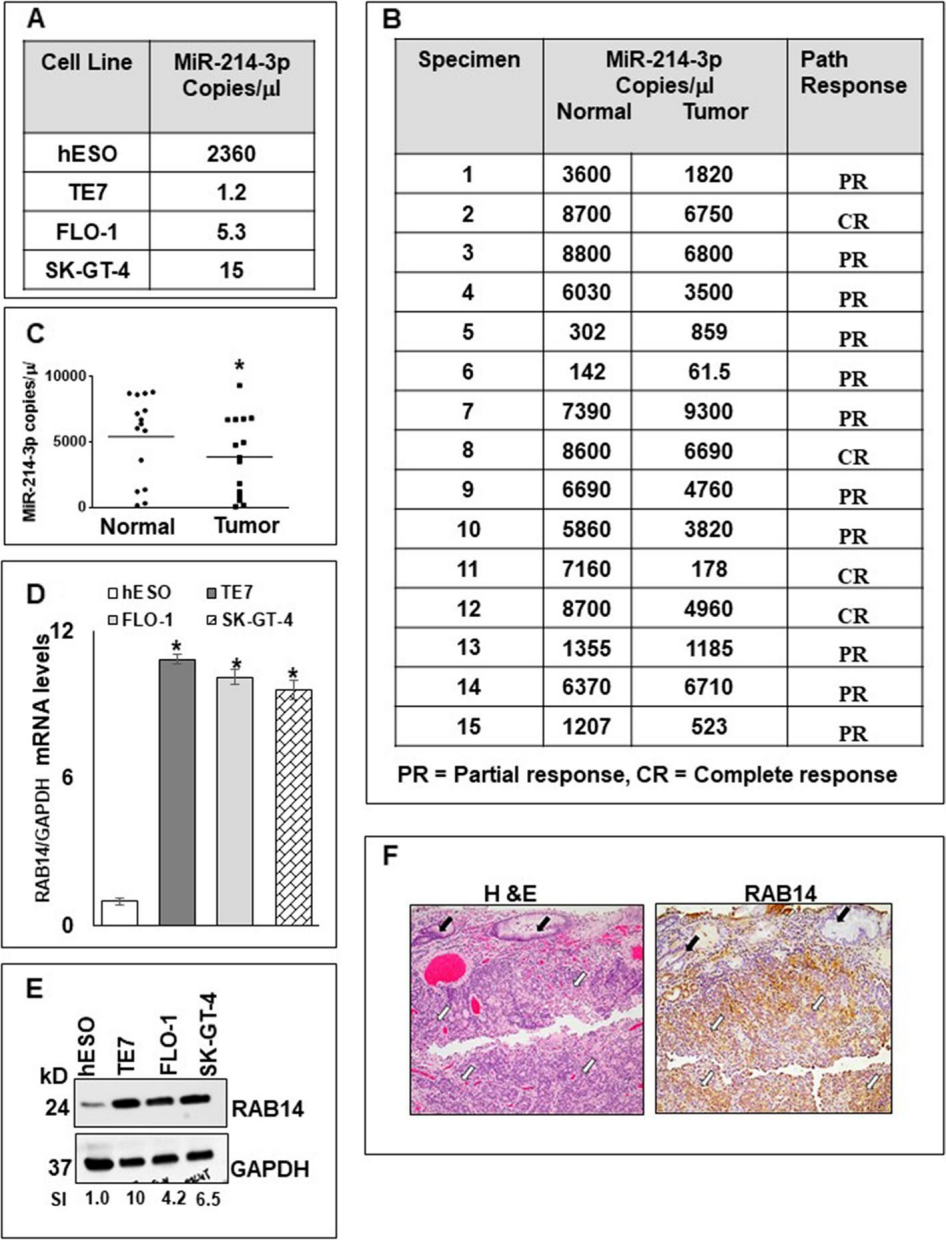
Basal levels of miR-214-3p and RAB14. Endogenous miR-214-3p expression levels measured as copy number per microliter by dd-PCR in (**A**) human esophageal epithelial (hESO) cells and esophageal cancer cell lines TE7, FLO-1 and SK-GT-4 and in (**B**) human esophageal tumor specimens with matched normal esophageal epithelial specimen, where PR = Partial response and CR = complete response. (**C**) Comparison of miR-214-3p expression detected by dd-PCR in the normal and tumor tissues in (B). * indicates statistical significance (*p* < 0.004). (**D**) Baseline RAB14 mRNA levels in hESO, TE7, FLO-1 and SK-GT-4 cell lines measured by q-PCR. Levels were normalized with GAPDH. Error bars represent mean ± S.D. Statistical significance is shown by *, where *p* < 0.05. **(E**) Endogenous RAB14 protein expression levels in the human esophageal cell lines. GAPDH was used as a loading control. The blot in this figure was cut prior to hybridization with antibody RAB14 and GAPDH. SI indicates relative RAB14 protein mean signal intensity measured using BioRAD Image Lab software. Signal intensity of the target protein (RAB14) is determined and is normalized by signal intensity of GAPDH. Relative signal intensity (SI) for target protein is calculated compare to hESO. **(F**) Representative images depicting hematoxylin and eosin (H&E) (left) and RAB14 staining (right) containing benign columnar epithelium and esophageal adenocarcinoma in human tumor samples. White arrows indicate invasive carcinoma. Black arrows indicate benign columnar epithelium. Scale bar 100 μm

Droplet digital PCR (ddPCR) was used to measure miR-214-3p copy numbers in human esophageal cancer cell lines as well as in matched malignant and non-malignant esophageal tissue from 15 patients with esophageal adenocarcinoma. As seen in Fig. 1A, expression of miR-214-3p is decreased between 2 and 3 log orders in 3 esophageal cancer cell lines (TE7, FLO-1, and SK-GT-4) compared to an esophageal epithelial cell line (hESO). Similarly, we observed that miR-214-3p expression was decreased in tumor samples compared to matched esophageal epithelium in 12 of 15 patients (Fig. 1B). Overall, expression of miR-214-3p was significantly decreased in the tumor samples compared to the esophageal epithelial samples (*p* = .004) (Fig. 1C). Interestingly, there was marked variability in miR-214-3p expression among the tumor samples, with copy numbers ranging from 61.5 to 9300 copies/μL. We did not observe any correlation between miR-214-3p levels in the tumor tissue and the degree of response (complete v. partial) to neoadjuvant chemoradiotherapy.

Conversely, as seen in Fig. 1D, levels of RAB14 mRNA are increased in the cancer cell lines by approximately 10-fold compared to hESO cells. This was associated with markedly enhanced RAB14 protein expression in the cancer cell lines compared to hESO cells assessed by Western blot (Fig. 1E). Figure 1F-left depicts hematoxylin and eosin (H&E) staining of a representative section containing benign columnar epithelium and esophageal adenocarcinoma form one of the patient tumor samples. IHC staining for RAB14 demonstrates positive staining in the invasive carcinoma, but negative staining in the benign epithelium (Fig. 1F-right).

### Modulating miR-214-3p levels leads to alterations in RAB14 protein expression and mRNA stability

**Fig. 2:**
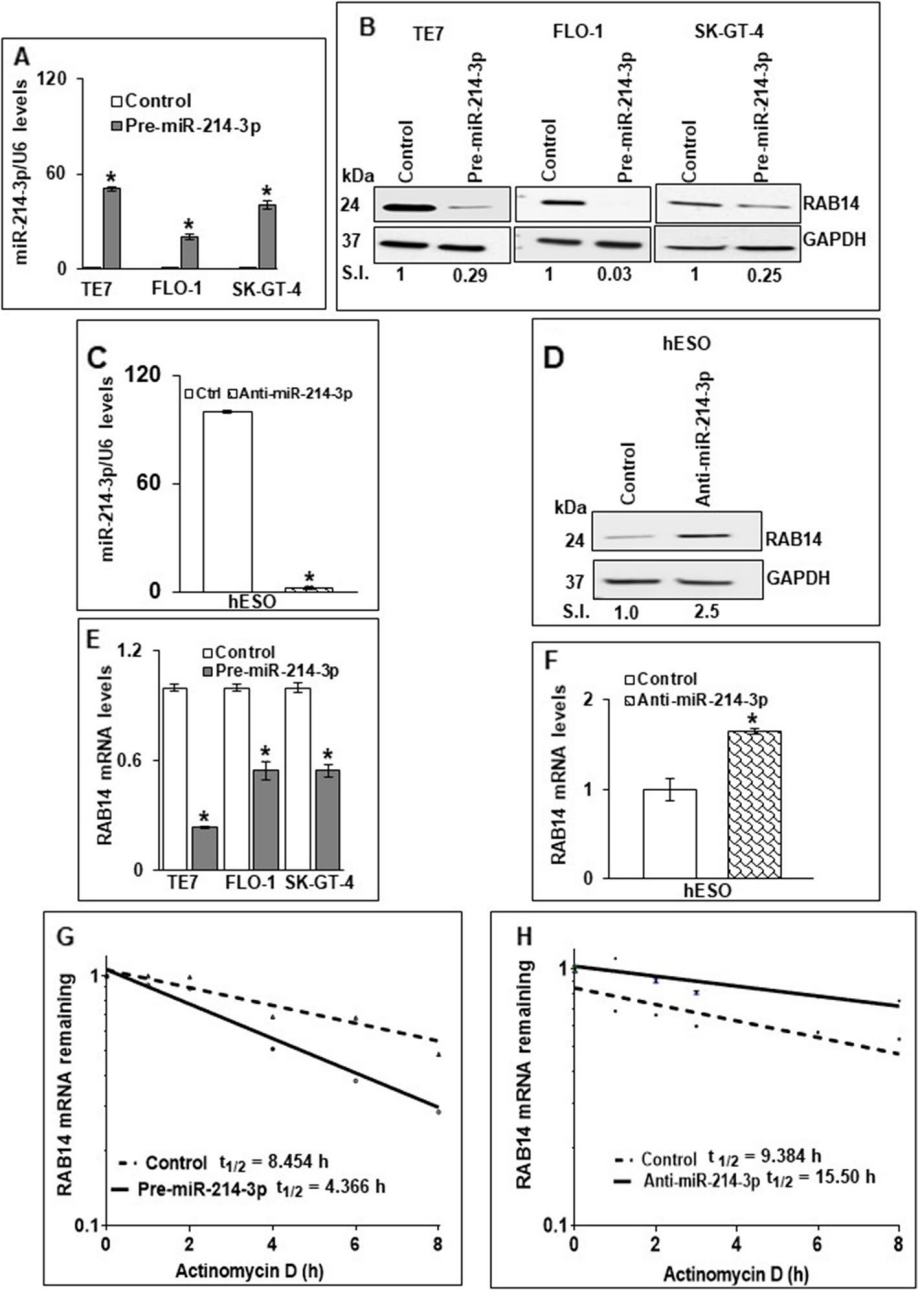
Effects of modulation of miR-214-3p on RAB14 protein and mRNA expression levels in human esophageal cells. (**A**) Levels of miR-214-3p in TE7, FLO-1 and SK-GT-4 cells transfected with pre-miR-214-3p (50 nM) as measured by q-PCR. (**B**) Changes in RAB14 protein expression after overexpressing miR-214-3p (50 nM) in TE7, FLO-1 and SK-GT-4 cells. Protein loading was assessed by GAPDH. The blots in this figure were cut prior to hybridization with antibody to RAB14 and GAPDH. Relative signal intensity was calculated as explained in Fig. 1E. (**C)** Levels of miR-214-3p after transfection of anti-miR-214-3p (25 nM) for 48 hrs in hESO cells as measured by q-PCR. Small nuclear U6 RNA was used as a control. Error bars represent mean ± S.D. * represents *p* < 0.05. **(D**) Effect of miR-214-3p silencing on RAB14 protein expression in hESO cells. hESO cells were transfected with anti-miR-214-3p (25 nM) for 48 hrs. Immunoblot was performed for RAB14 protein expression and GAPDH was used as a loading control. The blot in this figure was cut prior to hybridization with antibody to RAB14 and GAPDH. Relative signal intensity was calculated as explained in Fig. 1E. (**E**) Changes in levels of RAB14 mRNA in TE7, FLO-1 and SK-GT-4 cells following transfection of pre-miR-214-3p (50 nM). (**F**) Levels of RAB14 mRNA in hESO cells after transfection of anti-miR-214-3p (25 nM). Levels of RAB14 mRNA were measured by q-PCR. GAPDH was concurrently amplified to serve as an internal control. Error bars represent mean ± S.D. * signifies statistical significance (*p* < 0.05). Stability of RAB14 mRNA in (**G**) FLO-1 cells following transfection of pre-miR-214-3p and in (**H**) hESO cells after silencing miR-214-3p. Total RNA was isolated at indicated time points after administration of Actinomycin D (4 μM) and the remaining levels of RAB14 mRNA were measured by q-PCR. Levels were normalized with GAPDH. The half-life was calculated from the first order equation t1/2 = ln2/k

Because basal levels of miR-214-3p are low in the cancer cell lines, transfection of pre-miR-214-3p into these cells was performed in order to assess the effects on RAB14 protein expression (Fig. 2A). Increased miR-214-3p expression was associated with a marked decrease in RAB14 protein expression in all 3 cancer cell lines (Fig. 2B). In reciprocal experiments, anti-miR-214-3p was employed to reduce miR-214-3p levels in hESO cells (Fig. 2C). As seen in Fig. 2D, following successful transfection of anti-miR-214-3p, RAB14 protein levels are markedly increased in hESO cells.

To investigate the mechanism by which miR-214-3p regulates RAB14 protein expression, levels of RAB14 mRNA were assessed following overexpression of pre-miR-214-3p in TE7, FLO-1, and SK-GT-4 cells, as well as following transfection of anti-miR-214-3p in hESO cells. As seen in Fig. 2E, transfection of pre-miR-214-3p was associated with a significant decrease in RAB14 mRNA levels in all 3 cancer cell lines. In hESO cells, reduction of miR-214-3p expression led to a significant increase in RAB14 mRNA levels (Fig. 2F).

Figure 2G and H depict stability of RAB14 mRNA following transfection of pre-miR-214-3p in FLO-1 cells and anti-miR-214-3p in hESO cells, respectively. In these experiments, 24 hours following transfection, cells are exposed to 4 μM Actinomycin D to prevent further transcription. Total cellular RNA is harvested at specified time points and levels of RAB14 mRNA are measured by q-PCR. As seen in Fig. 2G, RAB14 mRNA is destabilized following pre-miR-214-3p transfection in FLO-1 cells, with a reduction in its half-life from 8.5 to 4.4 hours. Conversely, the stability curve in Fig. 2H demonstrate enhanced stability of RAB14 mRNA following silencing of miR-214-3p in hESO cells, with an increase in half-life from 9.4 to 15.5 hours.

### MiR-214-3p binds to RAB14 mRNA

**Fig. 3:**
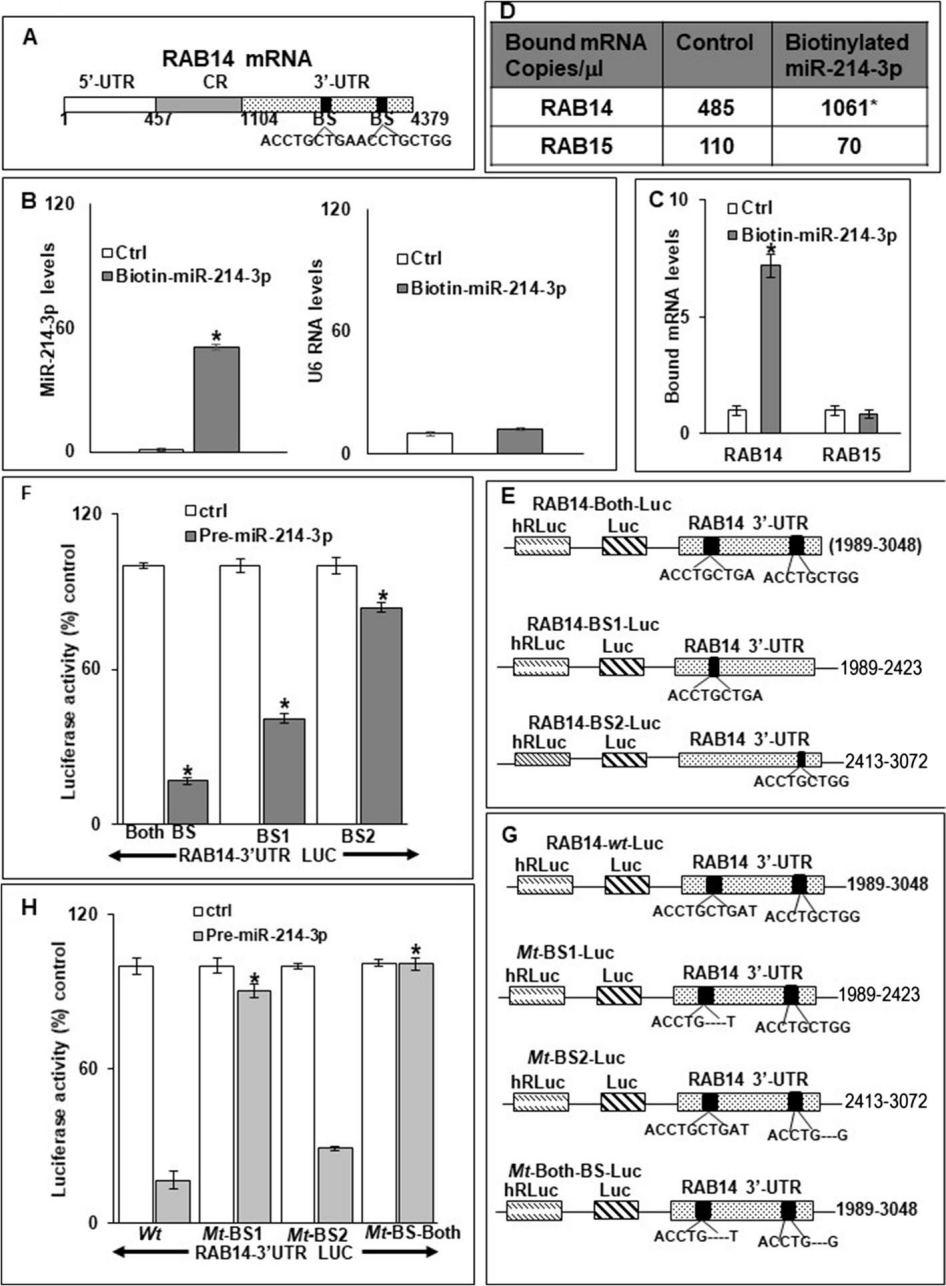
Association of miR-214-3p with RAB14 mRNA. (**A**) Schematic representation of RAB14 mRNA. (■) indicates predicted binding sites (BS) for miR-214-3p in the 3-UTR. (B) Levels of miR-214-3p (left) and U6 RNA (right) following transfection of 50 nM biotinylated-miR-214-3p (5′ ACAGCAGGCACAGACAGGCAGU 3’Bi) or a control miR for 48 h in TE7 cells, as measured by q-PCR. (**C**) Binding of miR-214-3p to mRNA encoding RAB14 and RAB15. Levels of RAB14 and RAB15 mRNA in the materials pulled down by biotinylated-miR-214-3p as measured by q-PCR. Representative bar diagram from three independent experiments is shown. Each set of experiments was done in triplicate. Error bars represent mean ± S.D. and * denotes statistical significance where *p* < 0.05. (**D**) Absolute levels (copy number) of RAB14 and RAB15 mRNAs in the materials pulled down by biotinylated-miR-214-3p as measured by dd-PCR. **(E-G**) Interaction of miR-214-3p with RAB14 mRNA. Schematic representation for RAB14 luciferase reporter constructs (**E & G**). RAB14 luciferase reporter constructs (**E**) containing either both or one of the predicted binding sites (BS1 or BS2). Reporter constructs (**G**) containing a mutation, i.e. deletion of 4 bases, in one or both predicted binding sites, (Mt-BS1, Mt-BS2, Mt-BS-both). Luciferase activity (**F & H**) in the RAB14 reporter constructs shown in E & G, following co-transfection with pre-miR-214-3p (50 nM) in TE7 cells. Cells transfected with control miR were considered to demonstrate 100% activity. Firefly luciferase activity was normalized to Renilla luciferase activity. Representative bar diagram from three separate sets of experiments is shown. Each set of experiments was done in triplicate. Error bars represent mean ± S.D. and * stands for statistically significant, *p* < 0.05. In H, * denotes significance relative to luciferase activity observed following transfection of pre-miR-214-3p with wild type, i.e. non-mutated, construct

We next sought to determine whether miR-214-3p interacted directly with RAB14 mRNA. As seen in Fig. 3A, there are two predicted miR-214-3p binding sites in the 3′ untranslated region (UTR) of RAB14 mRNA. Following successful transfection of biotin-labeled miR-214-3p or a biotin-labelled control miR into TE7 cells, cell lysates were exposed to streptavidin-coated beads (Fig. 3B). RNA was harvested from the pull-down material and amplified with probes for RAB14 and RAB15, employed as a control, by q-PCR. As seen in Fig. 3C, levels of RAB14 mRNA were markedly elevated in the pull-down material isolated from TE7 cells transfected with biotin-labeled miR-214-3p compared to control transfection, whereas no significant differences were seen in the levels of RAB15 between the samples. These findings were confirmed with ddPCR analysis of RAB14 and RAB15 mRNA levels in the pull-down materials (Fig. 3D).

In order to determine if both potential binding sites in RAB14 mRNA were being utilized for binding with miR-214-3p, the full-length RAB14 3’UTR containing both potential binding sites, as well as two fragments of the 3’UTR, each containing 1 potential binding site, were PCR amplified and separately sub-cloned into pmir-GLO dual-luciferase miRNA target expression vectors (Fig. 3E). Following co-transfection with pre-miR-214-3p into TE7 cells, there was an approximately 80% reduction in luciferase activity with the full length 3’UTR construct compared to control transfection. Co-transfection with the construct containing binding site 1 (BS1) resulted in an approximately 60% decrease in luciferase activity, while co-transfection with the construct containing binding site 2 (BS2) resulted in an approximately 10% decrease in luciferase activity compared to control (Fig. 3F).

To further substantiate the role of each potential binding site in mediating the observed effect, site-directed mutagenesis was performed to delete four bases in the seed sequence of each binding site individually, as well as in both binding sites (Fig. 3G). In agreement with the above results, co-transfection of the construct containing a mutation of binding site 2 (Mt-BS2) with pre-miR-214-3p had no significant effect on the reduction in luciferase activity seen following co-transfection of the unmutated construct containing both sites (Wt) with pre-miR-214-3p (Fig. 3H). Conversely, co-transfection of the construct containing a mutation of binding site 1(Mt-BS1) with pre-miR-214-3p significantly abrogated the reduction in luciferase activity seen following co-transfection of the unmutated (Wt) construct. Finally, mutation of both binding sites (Mt-BS-Both) completely eliminated the decrement in luciferase activity seen following co-transfection with the unmutated (Wt) construct. This suggests that both binding sites are required to achieve optimal efficacy, although binding site 1 is likely more critical.

### Overexpression of miR-214-3p decreases migration and invasion of esophageal cancer cells

**Fig. 4:**
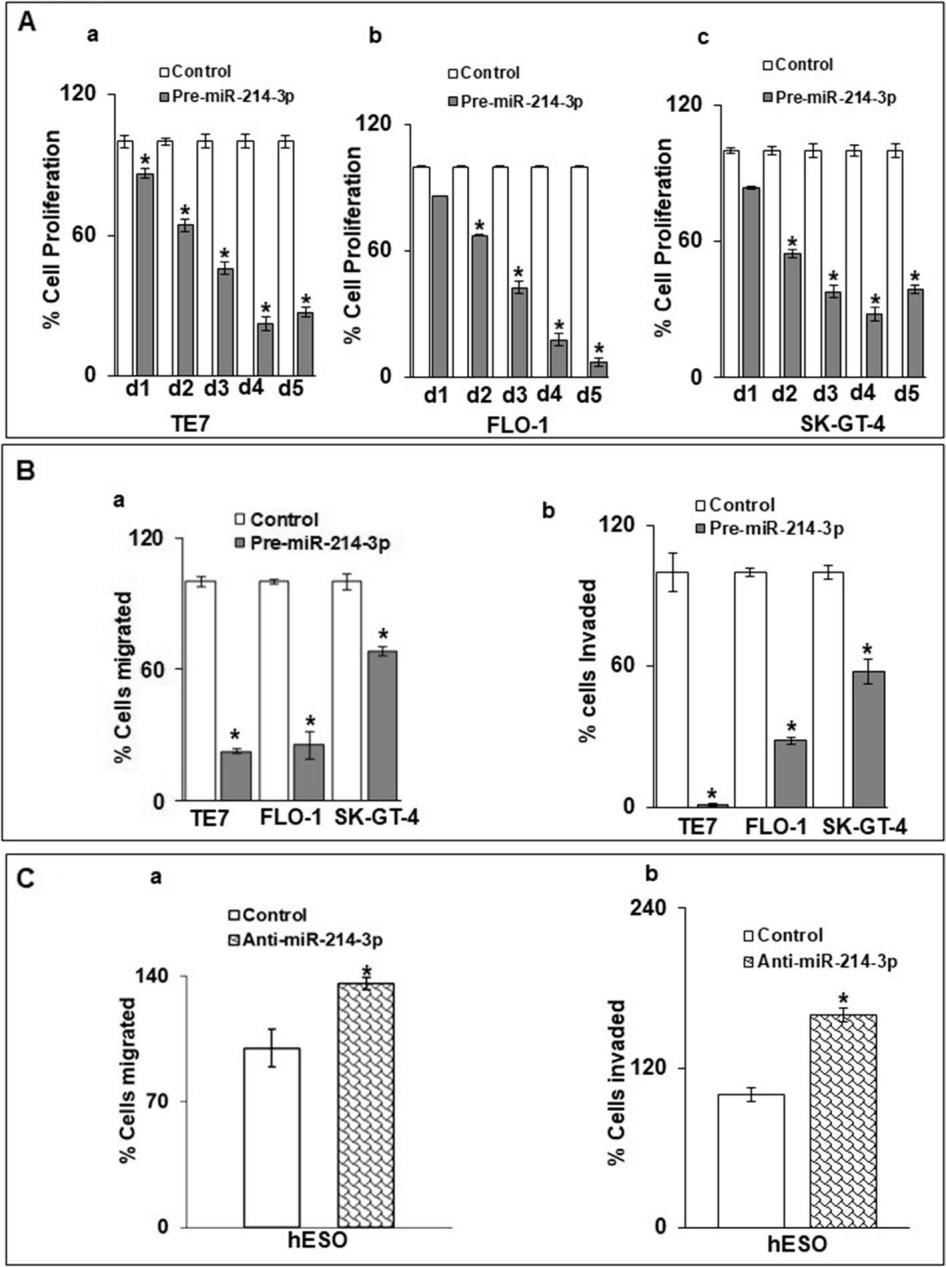
Effect of miR-214-3p on cellular proliferation, migration and invasion. **(A)** Cell proliferation (MTT) assay following transfection of pre-miR-214-3p in **a)**TE7, **b)** FLO-1, and **c)** SK-GT-4 cells. **B.** Cells (TE7,FLO-1 and SK-GT4) were transfected for 48 hours with pre-miR-214-3p (50 nM), followed by performance of (**a**) migration and (**b**) invasion assays in real time using XCELLigence-RTCA system. Changes in **(C-a)** migration and **(C-b**) invasion in hESO cells after silencing miR-214-3p. Cells were transfected with anti-miR-214-3p (25 nM) for 48 h prior to performance of the assay. Error bars represents ± S.D. and statistical significance is indicated by * (*p* < 0.05). Representative bar graphs for three independent experiments are shown

Based on previous reports documenting the ability of RAB14 to modulate cellular proliferation, migration, and invasiveness, we examined these phenotypes in TE7, FLO-1, and SK-GT-4 cells following forced expression of miR-214-3p [13–15]. We observed a statistically significant decrease in cellular proliferation in all three cell lines by 48 hours following pre-miR-214-3p transfection (Fig. 4A). In order to assess migration and invasion in real time, we utilized the xCELLigence RTCA system. As seen in Fig. 4B-a, cellular migration was significantly decreased in all three cell lines following miR-214-3p overexpression compared to control. Invasiveness was even more markedly impaired, with an observed decrease of approximately 90% in TE7 cells. (Fig. 4B-b) Interestingly, in complementary experiments, we found that silencing miR-214-3p in hESO cells resulted in significant increases in both the migration and invasion capacity of these cells compared to control (Fig. 4C).

**Fig. 5:**
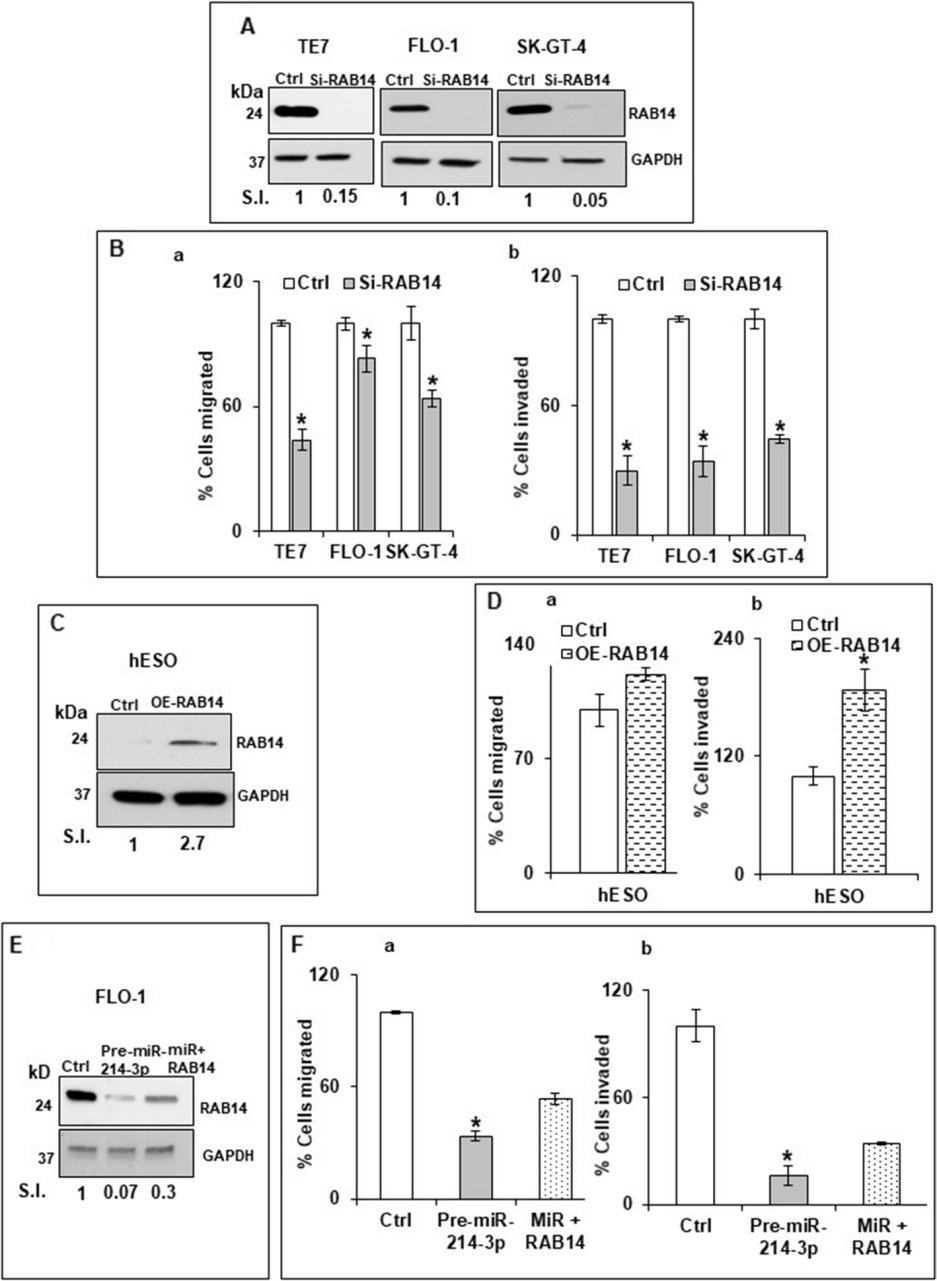
Effect of RAB14 on cellular migration and invasion. (**A**) Changes in levels of RAB14 protein in TE7, FLO-1, and SK-GT-4 cells following transfection of 80 pM RAB14-siRNA. The blots in this figure were cut prior to hybridization with antibody to RAB14 and GAPDH. Relative signal intensity was calculated as explained in Fig. 1E. (**B**) Migration and invasion assays in real time. Following silencing of RAB14 in TE7, FLO-1, and SK-GT-4 cells as described in (A), migration (**a**) and invasion **(b**) assays using XCELLigence-RTCA system were performed. Representative bar diagrams for three biological and technical replicates are shown. * denotes statistical significance (*p* < 0.05). (**C)** Changes in levels of RAB14 in hESO cells following transfection of 2.5 μg of a RAB14 expression plasmid. The blot in this figure was cut prior to hybridization with antibody to RAB14 and GAPDH. **(D**) Migration (**a**) and invasion (**b**) assays in real time using XCELLigence RTCA system were performed following overexpression of RAB14 in hESO cells as described in (C). Representative bar diagrams are shown. * denotes statistical significance (*p* < 0.05). (**E**) Following overexpression of pre-miR-214-3p (50 nM) (middle lane) or pre-miR-214-3p followed by 2.5 μg of RAB14 expression plasmid (last lane), RAB14 levels were measured by immunoblot and compared to control miR transfection. Relative signal intensity was calculated as explained in Fig. 1E. The blot in this figure was cut prior to hybridization with antibody to RAB14 and GAPDH. (**F**) Migration (**a**) and invasion (**b)** assays were performed in the samples explained in E. Mean ± S.D. is shown and statistical significance is indicated by * (*p* < 0.05)

In order to show that these effects on migration and invasion seen after miR-214-3p manipulation were mediated by changes in RAB14 expression, these experiments were repeated following RAB14 silencing in the cancer cell lines and RAB14 overexpression in hESO cells. Robust RAB14 silencing in all three cell lines was achieved following transfection with si-RAB14 (Fig. 5A). Although to a slightly less extent than seen following miR-214-3p overexpression, silencing of RAB14 also resulted in significantly decreased migration and invasiveness in all three cell lines (Fig. 5B). As seen in Fig. 5C-D, following successful overexpression of RAB14 in hESO cells, both migration and invasiveness were increased in these cells, with a statistically significant increase observed for invasiveness.

As a final step in this analysis, we examined whether re-introduction of RAB14 could partially offset the observed decrease in migration and invasiveness seen following miR-214-3p overexpression. In this experiment, 48 hours following successful overexpression of miR-214-3p in FLO-1 cells, the cells were transfected with the RAB14-expressing plasmid. As depicted in Fig. 5E, this results in partial restoration of RAB14 expression in these cells. This degree of restoration of RAB14 expression is associated with partial recovery of the migration and invasion capabilities of these cells (Fig. 5F).

### Overexpression of miR-214-3p leads to decreased tumor growth in a murine subcutaneous tumor model

**Fig. 6:**
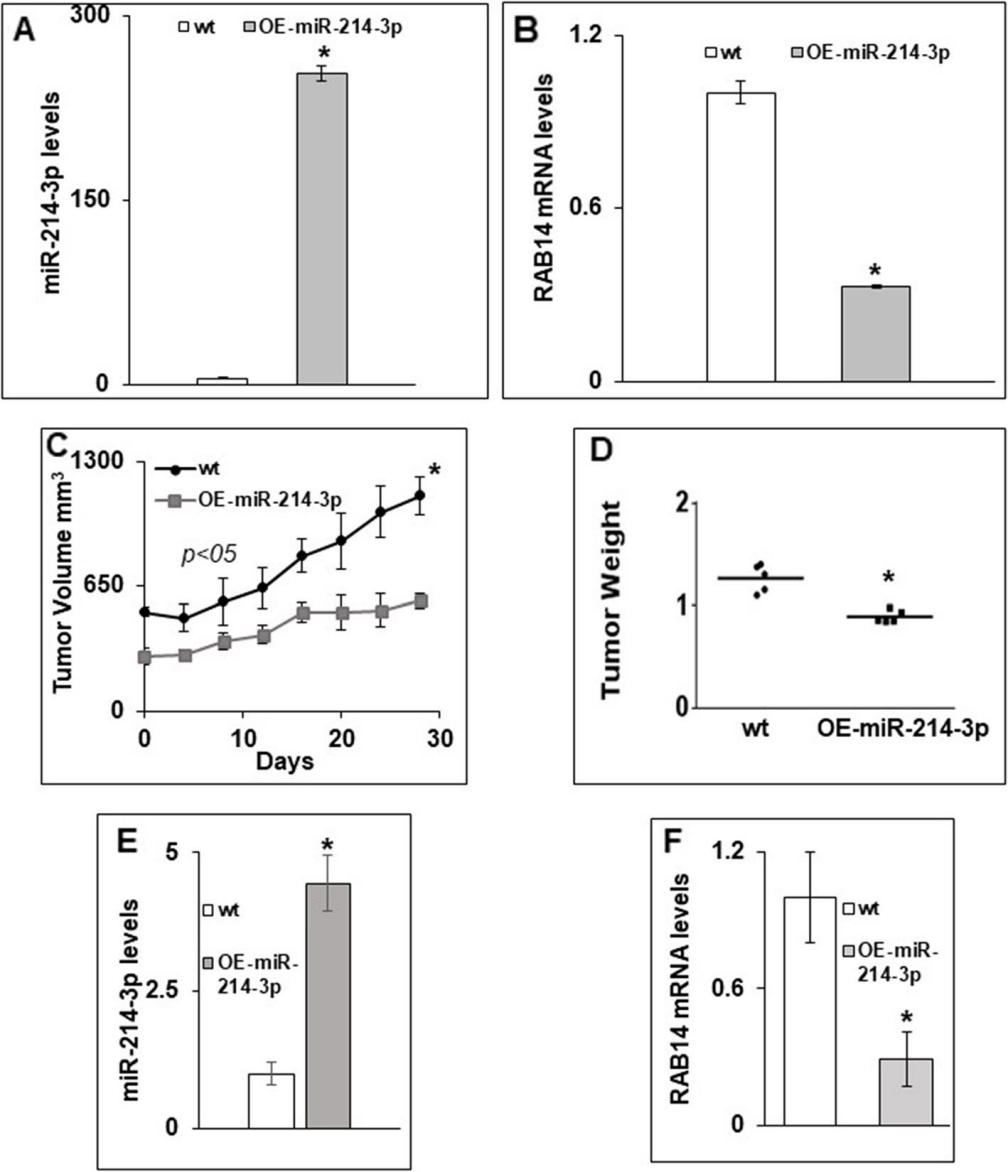
(**A**) Levels of miR-214-3p in FLO-1 cells that stably overexpress miR-214-3p compared to wild- type (wt) FLO-1 cells. (**B**) Levels of RAB14 mRNA in the cells mentioned in (A). To generate stably expressing clones, FLO-1 cells were transduced for 48 hours with a lentiviral-miR-214-3p construct and single clones were selected using puromycin (1 μg/ml) as a selection marker. Representative bar diagrams from three separate set of experiments are shown. Each set of experiments was done in triplicate. Statistical significance is indicated by * (*p* < 0.05). Error bars represent mean ± S.D. (**C-F)** Effect of miR-214-3p in-vivo. Wt and miR-214-3p stably overexpressing FLO-1 cells were subcutaneously injected in NRG mice. Tumor volume (**C**), and tumor weights **(D)** were measured in mice for 30 days, where day 0 indicates average tumor volume for mice in wt group measured ~ 500 mm3. Levels of miR-214-3p **(E)** and levels of RAB14 mRNA (**F**) were measured in tumors generated from FLO-1 cells stably expressing miR-214-3p harvested at day 30. Statistical significance is indicated by * (*p* < 0.05)

To assess the ability of miR-214-3p expression to mediate an anti-neoplastic effect in vivo*,* we generated a FLO-1 cell line that stably expresses miR-214-3p using a lentiviral transfection system. As seen in Fig. 6A-B, these engineered cells express significantly more miR-214-3p and significantly less RAB14 mRNA, than wild-type FLO-1cells. Using an NRG mouse subcutaneous tumor model, we found that FLO-1 cells stably expressing miR-214-3p grew significantly slower than wild-type FLO-1 cells (Fig. 6C). Differences in tumor volume between the groups were statistically significant at all measured time points. In addition, there was a significant decrease in the weights of tumors harvested at various time points following implantation from mice bearing FLO-1 tumors stably expressing miR-214-3p compared to those bearing wild-type tumors. A representative comparison of tumor weights harvested at 30 days following implantation is depicted in Fig. 6D. Finally, these tumors were digested and RNA isolated for assessment of intratumoral miR-214-3p and RAB14 mRNA expression. Similar to the results seen in the cultured cells, tumors generated from FLO-1 cells stably expressing miR-214-3p express significantly more miR-214-3p and significantly less RAB14 mRNA than tumors generated from wild-type FLO-1 cells out to 30 days from implantation (Fig. 6E-F).

## Discussion

Our findings indicate that miR-214-3p is markedly downregulated in multiple esophageal cancer cell lines, including both adenocarcinoma and squamous cell cancer cells, compared to esophageal epithelial cells. In addition, we found that expression of miR-214-3p is decreased in human esophageal cancer specimens compared to matched esophageal epithelial cells. Conversely, expression of RAB14 mRNA and protein were markedly upregulated in the esophageal cancer lines compared to hESO cells. Forced expression of miR-214-3p in these cells resulted in marked decreases in RAB14 mRNA and protein expression. In reciprocal experiments, silencing miR-214-3p in hESO cells resulted in increased RAB14 mRNA and protein levels. Direct interaction between miR-214-3p and its predicted binding sites in RAB14 mRNA was confirmed by biotinylated RNA-pull down assays and luciferase reporter constructs. Mechanistically, binding of miR-214-3p to RAB14 mRNA results in decreased RAB14 mRNA stability. Overexpression of miR-214-3p in esophageal cancer cell lines led to a marked decrease in the migration and invasion capabilities of these cells. These findings were replicated following silencing of RAB14, and could be partially reversed by RAB14 rescue following miR-214-3p overexpression. Finally, FLO-1 cells stably expressing miR-214-3p demonstrated decreased growth in a murine subcutaneous tumor model compared to wild-type FLO-1 tumors.

RAB14 is a member the RAB11 GTPase sub-family, which are components of endosomes that mediate the recycling of various proteins to and from the plasma membrane [13]. Endosomal recycling has been recognized as a critical regulatory element involved in cell motility [14]. In one such endosomal pathway mediated by RAB14, the protease ADAM10 is transported to the plasma membrane where it cleaved N-cadherin, thus promoting motility through decreased cell-cell adhesion [15]. In another endosomal pathway, phosphorylation of the RAB-coupling protein by LMTK3 enabled RAB14-dependent transport of the receptor tyrosine kinase EphA2 to the cell membrane where it mediated cell-cell repulsion [16]. Our results are the first to describe a role for RAB14 in esophageal cancer and consistent with previous reports of RAB14 affecting cellular migration and invasiveness in other malignancies. Overexpression of RAB14 has been demonstrated in pancreatic cancer and was corelated with poor prognosis [17]. In this study, silencing RAB14 resulted in decreased cellular proliferation and invasion, as well as increased sensitivity to gemcitabine, potentially through regulation of Bcl-2 expression. In bladder cancer, RAB14 overexpression was correlated with advanced stage and poor prognosis and was shown to activate the MAPK1/MAPK8 signaling pathway to enhance cellular migration and invasion [18]. RAB14 recycling by CHML was shown to enhance cellular migration and invasion through improved trafficking of regulators of metastasis such as Mucin 13 and CD44 to the cell membrane in hepatocellular cancer [19].

Similarly, these results support prior findings describing the role of miR-214-3p in promoting cellular migration and invasion. MiR-214-3p has been shown to be markedly downregulated in endometrial cancer specimens and cell lines [20]. In this study, overexpression of miR-214-3p in these cells inhibited cell migration and invasion, as well as epithelial-to-mesenchymal transition, by targeting TWIST-1. MiR-214-3p has also been shown to be downregulated in FGFR-amplified lung cancer and its overexpression was found to inhibit proliferation, migration, and invasion of lung cancer cells by targeting FGFR1 [21]. Furthermore, in this study, overexpression of miR-214-3p was found to exhibit synergistic effects with the FGFR-1 inhibitor AZD4547 in terms of decreasing tumor growth in both in vitro and in vivo models.

Finally, these data further contribute to the understanding of the impact of the downregulation of miR-214-3p on the development and progression of esophageal cancer. Lu and colleagues have shown that miR-214-3p was significantly downregulated in esophageal squamous cell cancer specimens compared to matched normal tissue [22]. Low miR-214-3p expression was associated with poor tumor differentiation and lymph node metastases in this study. Furthermore, overexpression of miR-214-3p resulted in decreased expression of GALNT7, a mediator of extracellular matrix interactions, leading to decreased migratory and invasive abilities of esophageal cancer cell lines. MiR-214-3p was also shown to be downregulated in a panel of 57 paired human esophageal squamous cell cancer samples and adjacent normal tissues [23]. In cell line experiments, overexpression of miR-214-3p resulted in decreased cellular proliferation and invasiveness by decreasing expression of CDC25B, a phosphatase with cell cycle regulatory properties. In another study, miR-214-3p was shown to be downregulated and beta-catenin upregulated in esophageal squamous cell cancer specimens compared to matched non-cancerous esophageal tissue [24]. Overexpression of miR-214-3p decreased beta-catenin protein expression, resulting in decreased cell growth and invasion.

Combined with our previous results showing that miR-214-3p is an important modulator of Cisplatin-induced apoptosis in esophageal cancer cells through regulation of survivin and CUG-BP1 expression, our current data provide strong support for the hypothesis that miR-214-3p functions as a key tumor suppressor in esophageal cancer cells. Our finding that miR-214-3p expression is decreased in the majority of human esophageal tumor specimens examined adds additional support to this hypothesis. Future studies should be directed at investigating whether miR-214-3p expression serves either a predictive or prognostic role in esophageal cancer patients and whether increasing intratumoral miR-214-3p expression results in therapeutic benefit using in vivo esophageal cancer models.

## Conclusions

Our results suggest that miR-214-3p is a key regulator of RAB14 expression and provide further evidence of the important tumor suppressive function of miR-214-3p in esophageal cancer cells.

## Supplementary Information

**Additional file 1: Fig. S1.** Original blots for Fig. 1E. Immunoblot for endogenous RAB14 protein expression levels in the human esophageal cell lines (top). GAPDH was used as a loading control (bottom). **Fig. S2.** Original blots for Fig. 2B and D Changes in RAB14 protein expression after overexpressing miR-214-3p in (A) TE7, (B) FLO-1 and (C) SK-GT-4 cells. RAB14 is shown in (top) blots and loading control GAPDH bottom blots. (D) Changes in RAB14 protein expression after inhibiting miR-214-3p in hESO cells (top). Protein loading was assessed by GAPDH (bottom). **Fig. S3.** Original blots for Fig. 5A, C and E Changes in RAB14 protein expression after silencing or over expressing RAB14. RAB14 (top) and GAPDH (bottom) expression in (A). TE7, (B). FLO-1 and (C). SK-GT-4 cells following silencing with si RNA. D. Changes in RAB14 protein expression after over expression of RAB14 Plasmid in hESO cells. Protein loading was assessed by GAPDH (bottom). E. Changes in RAB14 protein expression (top), in control (first lane), after overexpressing pre-miR-214-3p only (middle lane) and with RAB14 plasmid and pre-miR-214-3p (last lane). Protein loading was assessed by GAPDH (bottom). The blot was developed on Bio-RAD Chemidoc imager.

## Data Availability

The data supporting the finding of this study are available within the article and are available from the corresponding authors on reasonable request.

## Abbreviations

RAB14: Ras-related Protein 14
MiRs: microRNAs
CUGBP1: CUG triplet repeat RNA binding protein 1
ddPCR: Droplet Digital PCR
RTCA: Real Time Cell Analysis
O.D.: Optical density
