# Supplementary material for: MiR-214-3p targets Ras-related protein 14 (RAB14) to inhibit cellular migration and invasion in esophageal Cancer cells
Source: BMC Cancer. 2022 Dec 5;22:1265. doi: 10.1186/s12885-022-10304-0 (PMC9721009; doi:10.1186/s12885-022-10304-0)
Supplement: Supplementary file 1 — Additional file 1: Fig. S1. Original blots for Fig. 1E. Immunoblot for endogenous RAB14 protein expression levels in the human esophageal cell lines (top). GAPDH was used as a loading control (bottom). Fig. S2. Original blots for Fig. 2B and D Changes in RAB14 protein expression after overexpressing miR-214-3p in (A) TE7, (B) FLO-1 and (C) SK-GT-4 cells. RAB14 is shown in (top) blots and loading control GAPDH bottom blots. (D) Changes in RAB14 protein expression after inhibiting miR-214-3p in hESO cells (top). Protein loading was assessed by GAPDH (bottom). Fig. S3. Original blots for Fig. 5A, C and E Changes in RAB14 protein expression after silencing or over expressing RAB14. RAB14 (top) and GAPDH (bottom) expression in (A). TE7, (B). FLO-1 and (C). SK-GT-4 cells following silencing with si RNA. D. Changes in RAB14 protein expression after over expression of RAB14 Plasmid in hESO cells. Protein loading was assessed by GAPDH (bottom). E. Changes in RAB14 protein expression (top), in control (first lane), after overexpressing pre-miR-214-3p only (middle lane) and with RAB14 plasmid and pre-miR-214-3p (last lane). Protein loading was assessed by GAPDH (bottom). The blot was developed on Bio-RAD Chemidoc imager. [file 12885_2022_10304_MOESM1_ESM.zip › Fig-S2C-Original blot for figure 2B-SK-GT-4.pdf]

Full unedited gel for figure 2B (SK-GT-4)

Fig.S2C

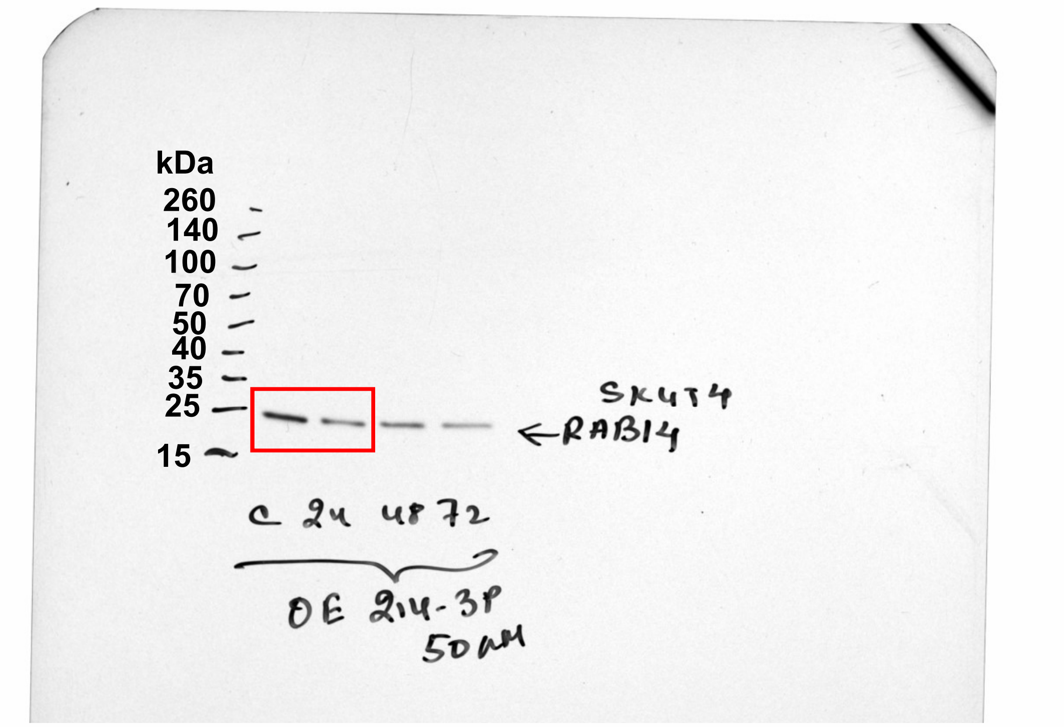

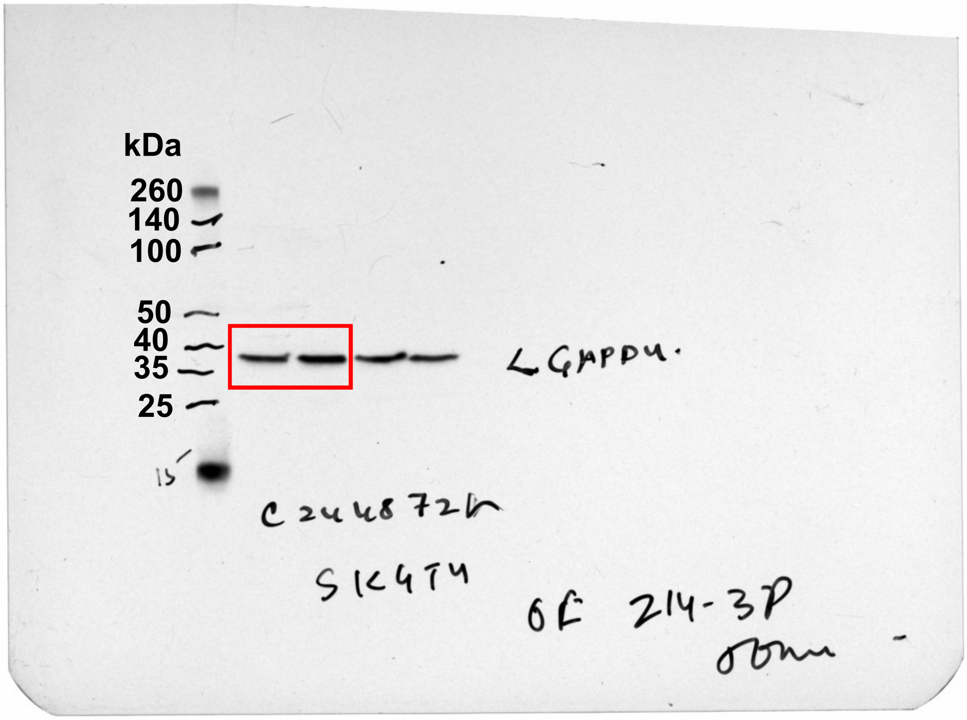

Fig.S2C. Original blot for figure 2B (SK-GT-4, right panel Changes in RAB14 protein expression following over expression of pre-miR-214-3p in SK-GT-4 cells (top). Protein loading was assessed by GAPDH (bottom.
